# Histone deacetylase inhibitor attenuates experimental fungal keratitis in mice

**DOI:** 10.1038/s41598-019-46361-y

**Published:** 2019-07-08

**Authors:** Xiaohua Li, Min Yuan, Ruijie Yin, Xiaohui Liu, Yu Zhang, Shengtao Sun, Lei Han, Shikun He

**Affiliations:** 1 Henan Provincial People’s Hospital, Zhengzhou, 450003 China; 2 Henan Eye Hospital, Henan Eye Institute, Henan Key Laboratory of Ophthalmology and Visual Science, Zhengzhou, 450003 China; 3 People’s Hospital of Zhengzhou University, Zhengzhou, 450003 China; 4 People’s Hospital of Henan University, Zhengzhou, 450003 China; 5 Departments of Pathology and Ophthalmology, Keck School of Medicine of the University of Southern California, Los Angeles, CA USA

**Keywords:** Corneal diseases, Chronic inflammation

## Abstract

Fungal keratitis is one of the leading causes of blindness of infected corneal diseases, but the pathogenesis of fungal keratitis is not fully understood and therefore the treatment of the disease by medication is still under investigation. In the current study, we sought to study the effect of HDAC inhibitor suberoylanilide hydroxamic acid (SAHA) on experimental fungal keratitis in mice. SAHA (25 mg/kg) (n = 30) or vehicle (DMSO) (n = 30) was delivered through intraperitoneal injection (IP) 24 hours after the fungal inoculation, and the same amount of SAHA injection or DMSO was followed at day 2. The expression of histone H3 (H3), acetylated histone H3 (AC-H3), histone deacetylase 1 (HDAC)1, tumor necrosis factor-α (TNFα), and Toll-like receptor 4 (TLR4) in surgically excised specimens from the patients and mice with fungal keratitis were detected by immunohistochemistry. The expression of mRNAs for Interleukin-1β (IL-1β), TNFα, and TLR4 were evaluated in the corneas of the mice with fungal infection and the control corneas by real-time PCR. The quantification of IL-1β and TNFα in the corneas of the mice with fungal infection was determined by ELISA. The inhibitory effect of SAHA on mice fungal keratitis was revealed by GMS and H&E staining. We found that the downregulation of histone acetylation and upregulation of HDAC1 expression were associated with the increased inflammation response in fungal keratitis not only in humans but also in experimental animals. SAHA was able to inhibit experimental fungal keratitis in mouse by suppressing TLR4 and inflammatory cytokines such as TNFα and IL-1β; the inhibition of HDAC may be a potential therapeutic approach for the treatment of fungal keratitis.

## Introduction

Fungal keratitis is one of the most prevalent corneal diseases and is also a major contributor to vision loss and blindness, especially in the developing countries. Among severe corneal infections, ~56% are due to fungal infections, as we previously reported^1^. The incidence of fungal keratitis in other developing countries is comparable with our finding^2,3^. The main risk factor for fungal keratitis is trauma, however, fungal keratitis has been observed after LASIK, corneal transplant, and abuse of topical corticosteroids^4^. It is known that numbers of factors contribute to the pathogenesis of fungal keratitis. Histological examination of the specimens from patients with fungal keratitis showed that inflammation induced by fungal organism is the major event in the local tissue. A variety of inflammatory cytokines participate in the initiation and progression of the disease; upregulation of tumor necrosis factor-α (TNFα), Interleukin-1β (IL-1β), Toll-like receptors (TLRs) and other inflammatory cytokines are demonstrated in fungal infected corneas^5,6^. Therefore, anti-fungal therapy is the common approach to control fungal induced inflammation. Unfortunately, medications for anti-fungal infection have limited efficacy and drug-resistance rapidly develops, suggesting that fungal keratitis is a complex disease and other mechanisms may participate in the pathogenesis of the disease.

Recent studies have demonstrated that epigenetic mechanisms can affect numerous pathologic conditions, including infectious diseases that are caused by microorganisms, angiogenesis, tumorigenesis, and tissue regeneration. Accumulating evidence suggests that epigenetic mechanisms, especially histone acetylation/deacetylation may play important roles in the pathogenesis of fungal infection. The genes that encode histone deacetylases (HDACs) are observed in fungal genomes^7^. By changing the histone modification tails, fungal pathogens are able to show differences in their virulence and drug resistance^8^. Importantly, HDAC inhibitors (HDACis) are also effective in controlling fungal infection to which there is acquired resistance clinically^9^. Brandão *et al*. demonstrated that HDAC inhibitors can alter the pivotal fungal virulence factors and fungal cell cycles^10^. Fungal growth is able to be inhibited up to 90% *in vitro* by HDACi^11,12^. HDACis also have been shown to be able to treat invasive aspergillosis^11^.

In terms of the application of HDACi to suppress inflammation, suberoylanilide hydroxamic acid (SAHA) has shown great promise as an anti-inflammation agent^13–15^. It exerts effects by targeting the major pre-inflammatory cytokines such as TNFα, IL-1β and TLR4^15,16^, Of note, SAHA is approved by FDA for treatment of T-cell lymphoma^17^. Previous publications have suggested that HDACs play an important role in the pathogenesis of fungal infection, however, the relevance of histone acetylation and fungal keratitis, especially the effect of HDAC inhibitor on fungal keratitis, have not been studied. In the current study, we investigated the expression of histone H3 (H3), acetylated histone H3 (AC-H3), HDAC1, TLR4, TNFα, and IL-1β in corneal specimens from patients with fungal keratitis and mice with experimental fungal keratitis. We also observed the inhibitory effect of SAHA on mice fungal keratitis.

## Results

### The increased expression of HDAC1 is associated with reduction of acetylated histone H3 in fungal keratitis

**Figure 1:**
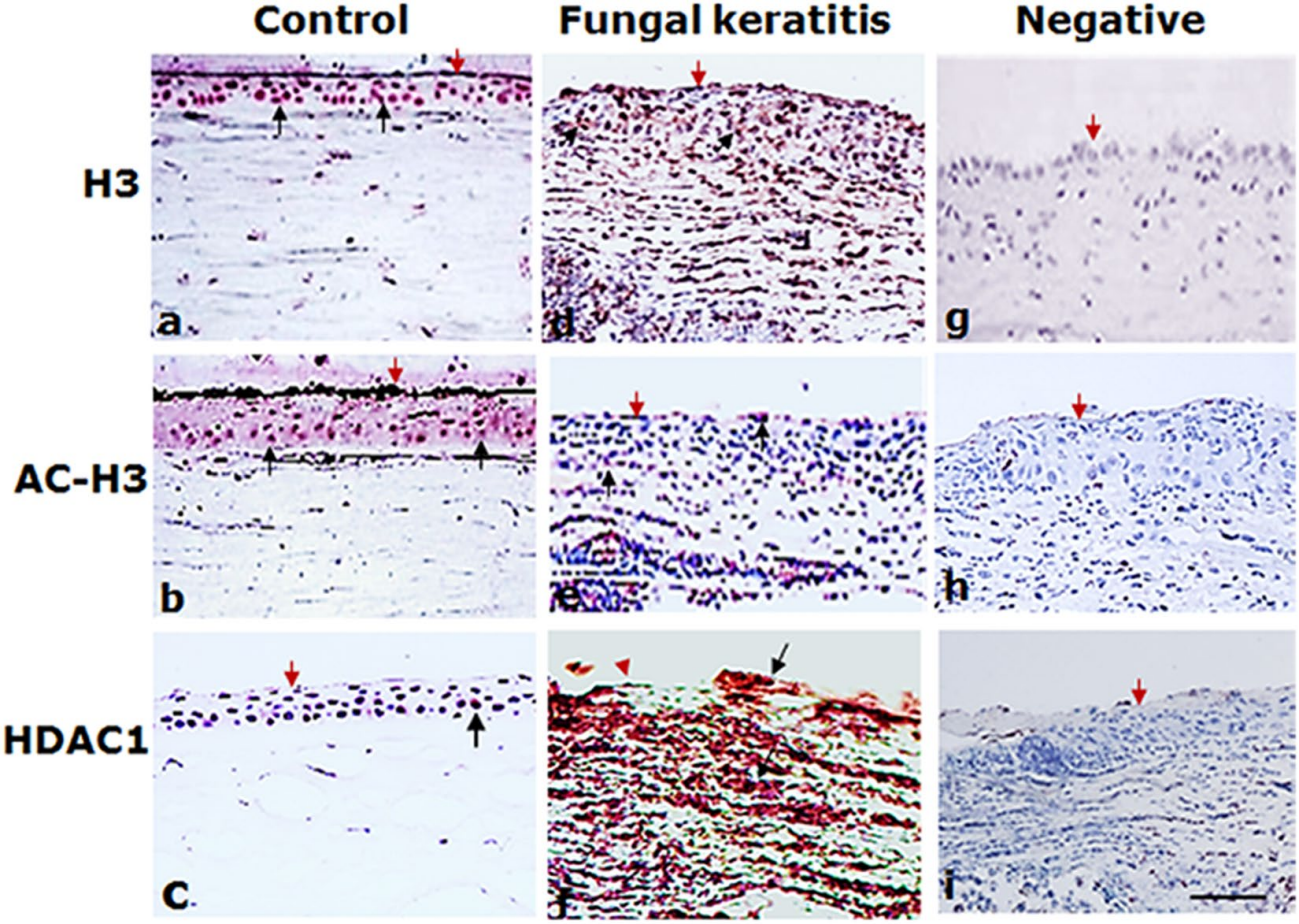
Immunohistochemical analysis of the expression of H3, AC-H3, HDAC1 in the specimens of patient with fungal keratitis. Red: positive staining; blue: hematoxylin contrast staining. The expression of HDAC1 was strikingly high in fungal infected corneal specimens; in contrast, the acetylated histone H3 was substantially reduced after fungal infection. No obviously difference is seen in the staining of H3 between specimens of fungal infected corneal tissues and control. The red arrows indicate the corneal surface and the black arrows represent focal positive staining. Scale bar: 50 µm. Original magnification, 200x.

**Figure 2:**
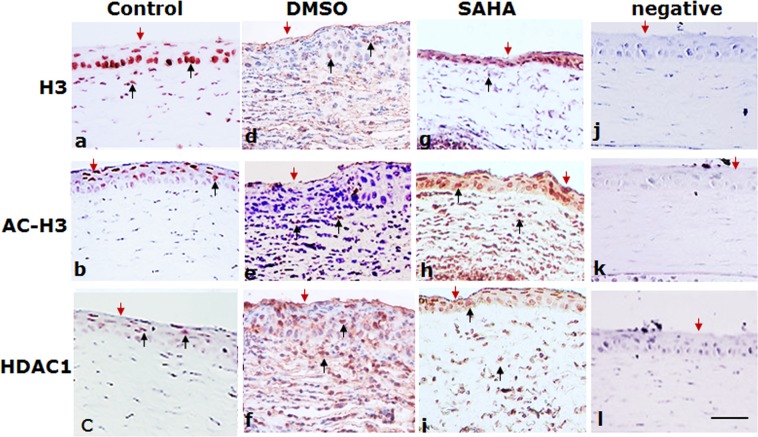
Immunohistochemical analysis of the expression of H3, AC-H3, HDAC1 in the specimens of the corneas of mice fungal keratitis. Red: positive staining; blue: hematoxylin contrast staining. The expression of HDAC1 was abundant in fungal infected corneal specimens of mice (**f**) in contrast, the acetylated histone H3 was substantially reduced after fungal infection. (**e**) The HDAC1 expression was inhibited after SAHA injection. No obvious difference is seen in the staining of H3 between specimens of fungal infected corneal tissue and control. Note the expression of AC-H3 was increased after SAHA injection. The red arrows indicate the corneal surface and the black arrows represent focal positive staining. Scale bar: 50 µm. Original magnification, 400x.

Reduced acetylated histone H3 (AC-H3) expression and increased HDAC1 expression in the corneal sections of human fungal keratitis and experimental fungal keratitis in mice compared with control were demonstrated by immunohistochemistry. The results showed that the expression of non-acetylated H3 was similar in both normal corneas and the specimens of fungal keratitis from both human and mice (Figs 1a,d and 2a,d,g). In the normal human corneas (Fig. 1b) and mice corneas (Fig. 2b), abundant AC-H3 expression was seen in the corneal epithelial cells, whereas AC-H3 was considerably reduced in the corneal specimens not only from patients with the keratitis but also from mice with experimental fungal keratitis (Figs 1a,e and 2e). In contrast, a strong expression of HDAC1 was demonstrated in cornea specimens from human and mice with fungal keratitis (Figs 1f and 2f). From this result, we therefore assumed that the downregulation of AC-H3 and the upregulation of HDAC1 in fungal keratitis might be implicated in the pathogenesis of the disease.

### Increased HDAC1 expression associated with enhanced expression of inflammatory cytokines

**Figure 3:**
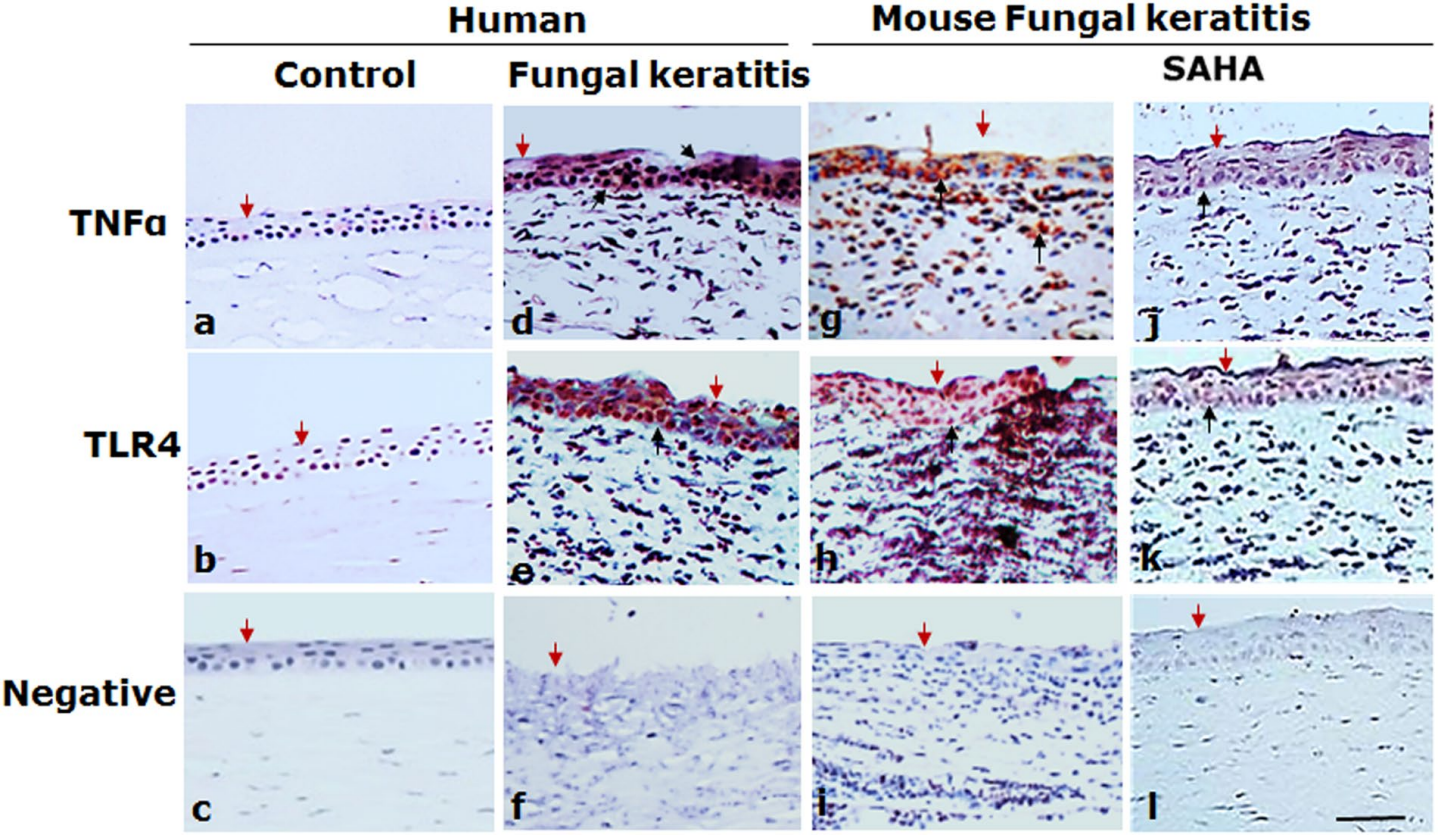
The expression of TNFα and TLR4 in the specimens of human fungal keratitis and the corneas of mice fungal keratitis. Red: positive staining; blue: hematoxylin contrast staining. Abundant immunoreactivity of TNFα and TLR4 is seen in fungal infected corneas of both human and experimental mice (DMSO injection); the expressions of TNFα and TLR4 in the corneas were inhibited by the application of SAHA in experimental mice. The red arrows indicate the corneal surface and the black arrows represent focal positive staining. Scale bar: 50 µm; Original magnification 300x.

**Figure 4:**
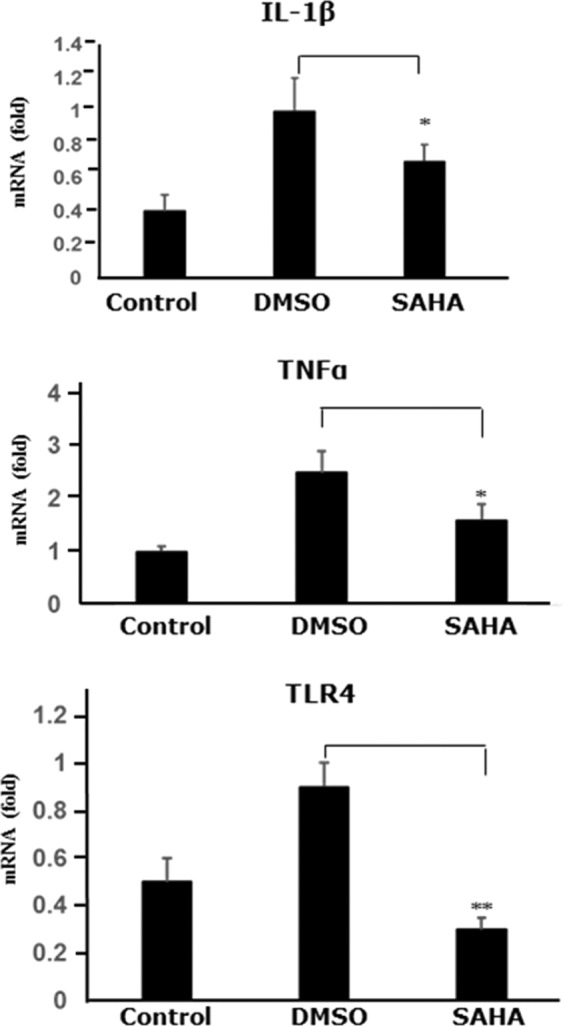
The effects of SAHA on the mRNA expression of IL-1β, TNFα and TLR4 in the corneal specimens of mice with experimental fungal keratitis. The total RNA was isolated from the mice corneas after the mice were sacrificed for the analysis of the mRNA expression of IL-1β, TNFα and TLR4 by real-time PCR. After treatment with SAHA, IL-1β and TNFα mRNA are reduced significantly compared with DMSO injection (*P < 0.05), while the inhibition of TLR4 is even greater (**0.01). The numbers of Y axial indicate the relative fold changes to GAPDH.

We demonstrated that the HDAC1 was highly expressed after fungal infection both in human and mice corneas, meanwhile we found that a substantial increase of immunoreactivity of TNFα and TLR4 was exhibited in cornea specimens from human with fungal keratitis and mice with experimental fungal keratitis (Fig. 3d,e,g,h). In addition, the mRNA expression of IL-1β, TNFα and TLR4 was significantly increased in the corneas with fungal infection, compared with the control group in which only DMSO injection was administrated (Fig. 4, P < 0.05).

### SAHA inhibits the expression of TNFα and TLR4

Immunohistochemistry showed that injection of SAHA inhibited the expression of TNFα and TLR4 (Fig. 3j,k), the intensity of the staining of TNFα and TLR4 was minor in SAHA treated corneal tissues (Fig. 3j,k), whereas in the control group, the expressions of TNFα and TLR4 were much stronger (Fig. 3g,h).

**Figure 5:**
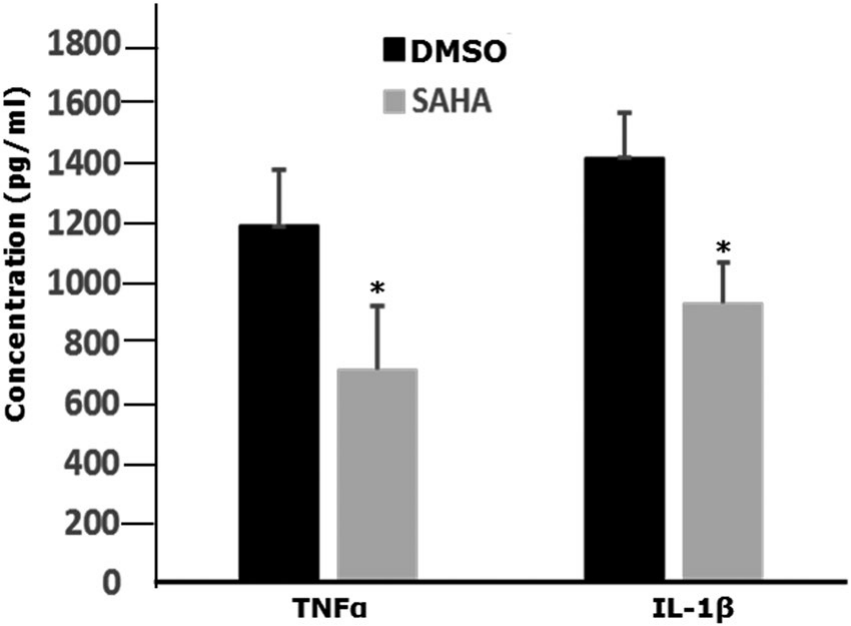
The effects of SAHA on the production of TNFα and IL-1β in corneas of mice with experimental fungal keratitis by ELISA. The protein obtained from the mice corneas of experimental fungal keratitis was subjected to ELISA assay, injection of SAHA significantly inhibited TNFα and IL-1β production compared with DMSO application (P < 0.05).

In support of the findings from immunohistochemistry, real-time PCR showed that SAHA injection significantly suppressed the mRNA expression of IL-1β, TNFα and TLR4 in fungal infected corneas compared with the injection of vehicle without SAHA (Fig. 4, P < 0.05). Consistent with the findings of the mRNA data, ELISA assay demonstrated that the production of IL-1β and TNFα were significantly reduced in the corneal tissues after SAHA injection compared with the injection of vehicle without SAHA (Fig. 5, P < 0.05).

### SAHA suppress experimental fungal keratitis

**Figure 6:**
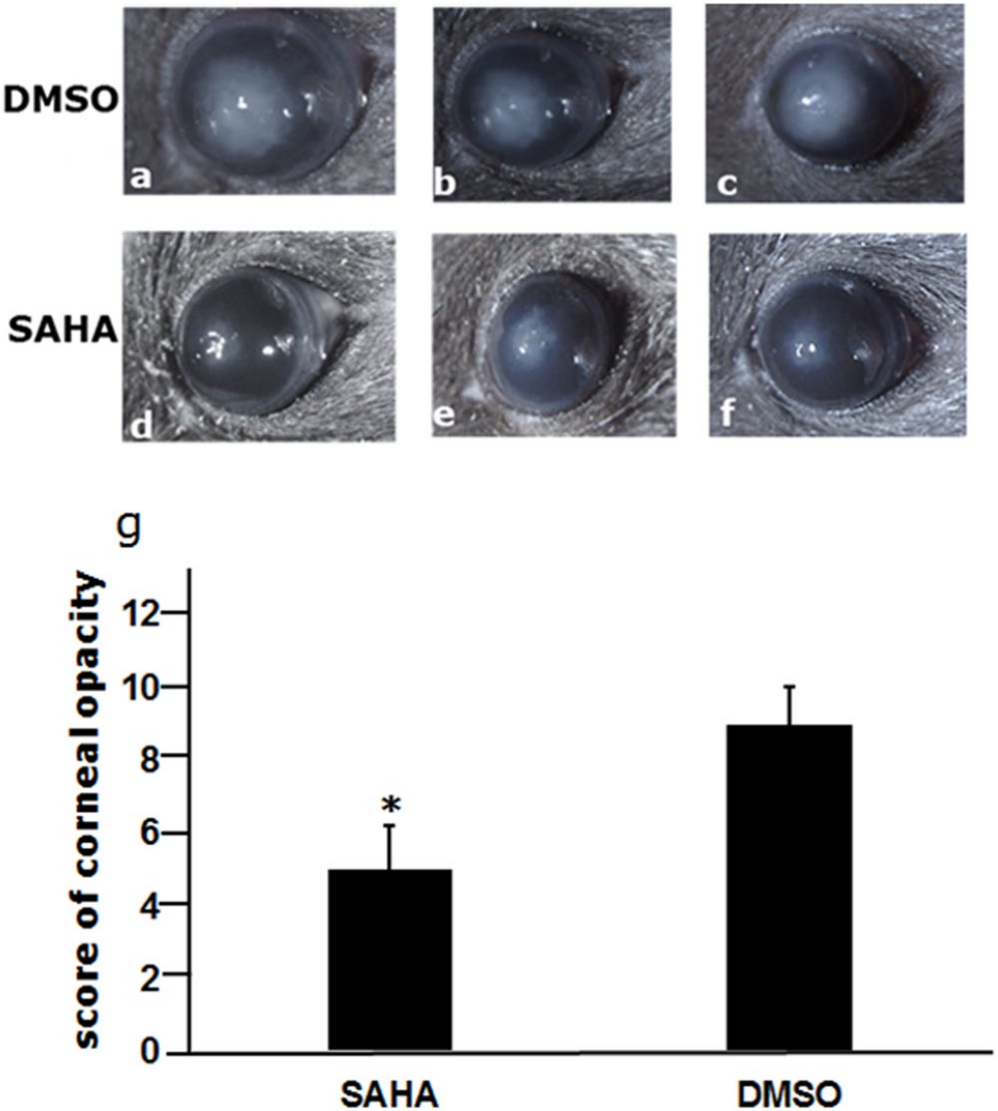
The effects of SAHA injection on the corneal opacity of experimental fungal keratitis in mice. The corneal opacity was considerably reduced in the mice corneas with SAHA injection (**a**–**c**) compared with DMSO application. (**d**–**f**) The inhibition of the corneal lesions by SAHA injection was significant (**g**), (P < 0.05), photos are representative.

Injection of SAHA provided a protective role for the mice with fungal inoculation; the corneal opacity caused by fungal infection in the cornea was reduced by SAHA injection compared with DMSO application (Fig. 6a–f), the inhibition was significant (Fig. 6g; P < 0.05).

**Figure 7:**
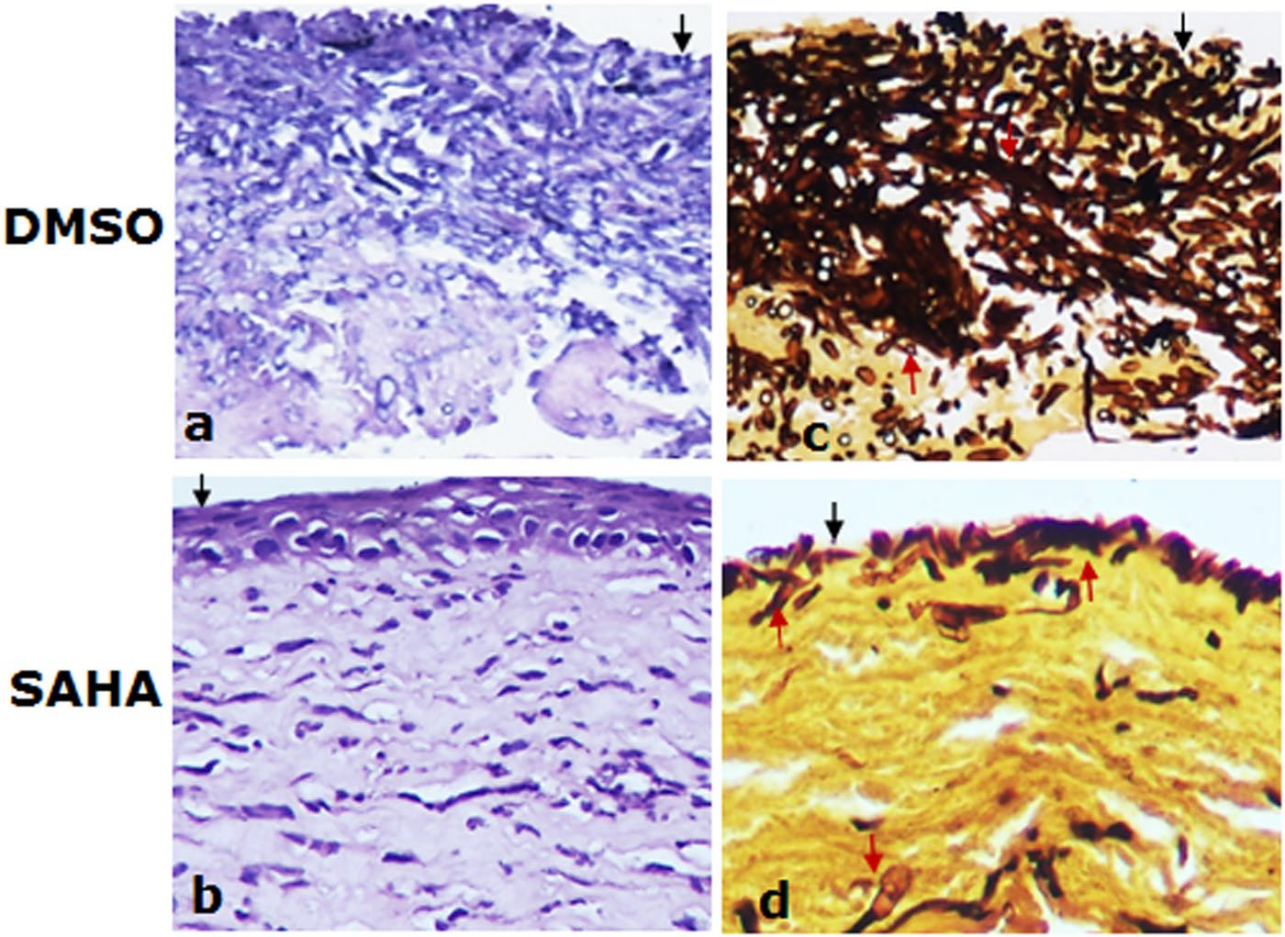
Injection of SAHA inhibited the fugal growth in the mice cornea. Fungal growth were inhibited in SAHA treated mice cornea as demonstrated by H&E (**g**,**h**), GMS staining (**i**,**j**) respectively. Dark brown stained fungal. The black arrows indicate the surface of the corneas and the red arrows represent fungal. Scale bar: 50 µm; Original magnification 300x.

Histological analysis showed that decreased infiltration of inflammatory cells was seen in SAHA treated cornea specimens (SAHA injection) compared with control (Fig. 7a,b). Importantly, the fungal growth in the cornea was suppressed by SAHA injection as showed by GMS staining (Fig. 7d), in contrast, abundant fungal growth could be seen in the group without SAHA application (Fig. 6c).

## Discussion

Post-translational modifications of histones, including histone acetylation and deacetylation, have been recognized as an important epigenetic mechanism in the regulation of gene expression. Histone acetylation and deacetylation are catalyzed by HATs and HDACs. Accumulating evidence suggests that reduced histone acetylation is associated with increased histone deacetylase (HDAC) and plays an important role in the pathogenesis of many complex human diseases^18–24^.

Notably, decreased histone acetylation can be restored or upregulated by the application of HDAC inhibitors (HDACis). Research has shown that HDACis can be used to treat numerous clinical diseases in human and animal models, including inflammation, angiogenesis, tumorigenesis, aging, degenerative diseases and regeneration^18–24^. Interestingly, inhibition of histone deacetylation as novel anti-fungal therapy has been shown to be an option in the treatment of fungal infection^25^, However, the effect of the HDAC inhibitors on the experimental fungal keratitis has not been reported.

In the current study, we found that HDAC1 was highly expressed in corneal specimens from human with fungal keratitis and mice with experimental fungal keratitis. In contrast, the acetylated histone3 (AC-H3) was considerably reduced in corneal specimens both from human with fungal keratitis and from mice with experimental fungal keratitis, indicating that there was a histone hypoacetylation in the fungal infected corneal tissue due to enhanced expression of HDAC1. Importantly, the decreased AC-H3 was restored after SAHA treatment, while the HDAC1 was significantly reduced in the corneas of mice with fungal keratitis with the application of HDAC inhibitor-SAHA. Indeed, the results suggest that histone acetylation and deacetylation are involved in the pathogenesis of fungal keratitis. In a previous study, the importance of the expression of HDAC1 in fungal virulence has been reported^11^. It has been demonstrated that by knocking down HDAC1 in embryonic stem cells, the proliferation of the cell is compromised due to the increased expression of the genes of p21 and p27^26^. In addition, numerous proteins in the control of fungal virulence are regulated by HDACs. Importantly, by knocking down HDAC gene, the growth of the fungal organism is inhibited^11,27–29^. Taken together, the data suggests that fungal infection is regulated by histone acetylation and deacetylation and the increased expression of HDAC1 and hypoacetylation may contribute to the pathogenesis of fungal keratitis.

Previous studies have shown that HDACi is a potential treatment candidate of fungal infection^30^. We further looked into the inhibitory effects of HDAC inhibitor on fungal keratitis in mice. It is known that the HDACi-SAHA have been approved by FDA for cancer therapy (cutaneous T-cell lymphoma) and several other human diseases for clinical trials^31^. SAHA and TSA are the most commonly used HDACis. The reason why we selected SAHA in our study is that SAHA exhibits a longer half-life than TSA, Therefore, in this study we investigated the effect of SAHA on fungal keratitis in a mouse model. Basimia *et al*. found that SAHA could inhibit the production of aflatoxin and fungal growth *in vitro*^32^. Our results showed that the fungal growth in the cornea of mice with fungal keratitis was suppressed after SAHA treatment as demonstrated by GMS staining; most importantly, the corneal opacity was reduced in the SAHA treated group compared with control group as showed in Fig. 6, suggesting that SAHA could protect mice from fungal infection.

It is well known that the TNFα and IL-1β are major pro-inflammatory cytokines induced by fungal infections^33–36^. TLR4 is upregulated in mice model of fungal keratitis and regulate fungal growth during fungal infection in cornea^5^. TLR4 activation lead to the upregulation of the expression of TNFα and IL-1β and other inflammatory cytokines^37^. In the current study, we found that there was abundant expression of TNFα and TLR4 in the specimens from patients with fungal keratitis and mice with experimental fungal keratitis. The accumulated evidence implicates that TLR4 and TNFα are important in the pathogenesis of fungal infection, hence, we investigated if SAHA affects fungal keratitis due to the inhibition of key cytokines-TNFα and the inactivation of TLR4. This was corroborated by results of real-time PCR at mRNA levels of TLR4, TNFα and IL-1β, as well as the ELISA results at protein levels of TNFα and IL-1β of the corneal specimens from mice with fungal keratitis. It was found that the expression of mRNA of TLR4, TNFα and IL-1β of the corneal specimens from mice with fungal keratitis was significantly suppressed after SAHA treatment compared with control, the production of TNFα and IL-1β was also inhibited with SAHA treatment as shown by ELISA assay. It is known that activation of TLRs including TLR4 is able to upregulate the downstream inflammatory cytokines such as TNFα and IL-1β.

TNFα is one of the strongest factors to induce inflammation and cell necrosis. We and other researchers^38^ demonstrated that HDACi can inhibit the expression of IL-1β and TNFα, therefore SAHA may protect the corneal cells from death induced by TNFα and limit the development of the corneal ulceration. Furthermore, it is found that FOXO3 is an anti-inflammatory factor^39^, SAHA can activate FOXO3 through inhibiting Akt signaling^40^. In addition, previous study show that NF-kB is suppressed by SAHA in cancer cells^41^. It is possible that one of important mechanisms of corneal ulcer reduction might be related to downregulation of inflammatory cytokines, especially TNFα and NF-ḳB, and the increased expression of FOXO3 induced by SAHA application.

HDACs plays an important role in the controlling of filamentous fungi growth and contributes to virulence via the regulation of variety of transcription factors and proteins^11,42,43^ Thus, HDACi represent an alternative to antifungals in the treatment of fungal infections. It has been previously shown that HDACi is effective either as monotherapy *in vitro*, or as adjunctive therapy against fungal infections^42,44,45^. Our results showed that the downregulation of histone acetylation and upregulation of HDAC1 expression were associated with the increased inflammatory response in fungal keratitis. SAHA was able to inhibit experimental fungal keratitis by suppressing TLR4 and inflammatory cytokines (TNFα, IL-1β) and inhibition of HDAC may be a potential therapeutic approach for the treatment of fungal keratitis.

Our study, to the best of our knowledge, is the first to investigate the effects of HDACi in a mouse model of fungal keratitis. The usage of HDACi in fungal keratitis may be a promising venue and alternative treatment to traditional antifungal therapy.

## Material and Methods

### Patients

The Institutional Review Board of the Henan Eye Institute approved our use of human corneal specimens. All procedures conformed to the Declaration of Helsinki for research involving human subjects. Informed consent have been obtained from all participants. The 10 corneal specimens were collected for retrospective histological analysis after surgery, which included penetrating keratoplasty, as well as evisceration and enucleation due to fungal infection. All surgeries were performed by corneal specialists at the Henan Eye Institute. Six normal corneal specimens (n = 6, 30–65 years; mean age: 41 years) were used as control.

### Mice fungal keratitis model

All mice were treated in accordance with the guidelines provided in the Association for Research in Vision and Ophthalmology statement for the Use of Animals in Ophthalmic and Vision Research and the cares and experimentation protocols was approved by the Ethical Committee of Experimental Animal Care and Use of Henan Eye Institute. 60 C57BL/6 mice (6–8 weeks) male mice were purchased from Model Animal Research Center of Nanjing University (Nanjing, China).

The mouse model of the fungal keratitis was developed according to a previously described method^46^. Briefly, fusarium solani cultures were obtained from microbiology lab of Henan Eye Institute. Mice were anesthetized by intraperitoneal injection of sodium pentobarbital (80 mg/kg.b.w.) (Sigma–Aldrich, St. Louis, MO), 1% tetracaine hydrochloride eye drops were used for local anesthetize. Before fungal inoculation, a 2 mm circular area in the central cornea was marked in the center corneas of the mice using a trephine under dissection microscope (Topcon OMS-90, Japanese), the marked corneas were cross scratched within circular area using a sterile scalpel blade (carbon steel, size 11, Shanghai Pudong Jinhuan Medical Products Co. Ltd., China), the depth of the scratch was exceeded Bowman’s membrane. A rough surface was created by applying of a bamboo toothpick (0.30 mm tip diameter, 1.10 mm tip length) in the scratched area of the cornea to create a rough surface (histological examination showed that the corneal epithelial cells were removed in the scratched areas). Afterward Fusarium solani was topically applied to the surface of the cornea using the tip of a sharpened bamboo stick for fungal inoculation. It was performed by the same experienced researcher. The criteria of establishment of the fungal keratitis in mice was adapted from previously described method^46^, the infection was located to the scratched area by clinical and histologic examination. SAHA (25 mg/kg; Sigma-Aldrich) (n = 30) or vehicle (DMSO, Sigma) (n = 30) was injected through IP 24 hours after the fungal inoculation and the same amount of SAHA injection or DMSO was followed at day 2. At day 3, the SAHA treated and control animals were sacrificed after the corneas of the mice were scored using slit lamp and/or photographed in the intact cornea using microscopes (Leica SteREO Discovery. V20). Corneal lesion was scored according to the previous publications^47,48^. With the aid of slit lamp by two experienced researchers. A grade of 0 to 4 was scored according to area of opacity, density of opacity, and surface regularity (Table 1). The corneas photography was analyzed by two researchers in the mask manner and un-agreed case was excluded from the result. The eyes were enucleated for histological analysis, immunohistochemistry staining, ELISA assay and real-time PCR. The final total numbers of mice used in the study was 60, and 60 eyes were scored and corneal photography was taken for each mouse. 36 corneas were used for histological analysis, Gomori methenamine silver (GMS) staining and immunohistochemistry, 24 corneas were used for real-time PCR and ELISA.

**Table 1:** Clinical scoring criteria.

Grade	Area of corneal opacity	Density of corneal opacity	Surface regularity
1	1–25%	slightly opacity of the cornea, relatively clear pupil and iris	slight irregularity of the lesion surface
2	26–50%	opacity of the corneal superficial layer, visible pupil and iris through the lesion	rough and lesion surface with some swelling
3	51–75%	uneven opacity of the whole corneal layer	Significant swelling, crater or serious descemetocele formation
4	76–100%	even and dense opacity	Perforation or descemetocele

### Histopathological examination

Corneal specimens (16 from human and 36 from mice) were fixed in 10% neutral buffered formaldehyde and embedded in paraffin. Paraffin sections of 3 µm thickness were stained with hematoxylin and eosin (H&E) staining for general histological analysis. Gomori Methenamine-Silver (GMS) staining was performed to identify hyphae and spores for fungal infection. All of the stained corneal sections were reviewed by two ocular pathologists under light microscopy to avoid bias. Histological changes in all layers of the cornea were observed. The presence of fungal infection was confirmed by the existence of hyphae and spores.

### Immunohistochemistry

The method for Immunohistochemistry was adapted as described before^49,50^ briefly, 16 human and 36 mice corneal specimens were fixed in 4% paraformaldehyde, embedded in paraffin, and cut into 3-µm-thick sections. The sections were then stained using immunohistochemical methods after deparaffinization and rehydration (phosphate-buffered saline (PBS) pH 7.4). Antigen retrieval was performed by immersing sections of tissue in citrate buffer (pH 6.0) for 15 minutes and blocked with 5% normal goat serum for 30 minutes. Specimens were washed three times with PBS after each step. Sections were incubated with anti-histone H3 (Abcam, Cambridge, MA), anti-acetylated histone H3 (Abcam, Cambridge, MA), anti-HDAC1 (abcam, Cambridge, MA), anti-TNFα (Santa Cruz Biotechnology, CA) and TLR4 (Abcam, Cambridge, MA), then followed by application of biotinylated secondary antibody (Vector Laboratories, Burlingame, CA), and streptavidin peroxidase. The immunoreactivity was visualized using a 3-amino-9-ethylcarbazole (AEC) kit (Zymed, South San Francisco, CA). Isotype-matched primary antibody was used as a negative control. Slides were counterstained with H&E staining and mounted with a glycerin gelatin mounting medium.

### RNA isolation and real-time PCR

4 corneas from each group (DMSO and SAHA group) of the mice for the application of real-time PCR, 8 corneas were collected from one experiment. The corneas were cut into two parts through exactly the middle of the corneas in which half of each cornea was used for real-time PCR and another half was used for ELISA assay. Total RNA was isolated from 4 mice corneas from each group, using RNeasy kit (QIAGEN, Valencia, CA, USA) according to the manufacturer’s instructions. The cDNA was synthesized from 1 μg of total RNA using AMV reverse transcriptase (QIAGEN, Germantown, MD), following the instruction of the manufacturer. The cDNA was subjected to quantitative PCR on a real-time reverse transcriptase PCR system (Life Technologies, Grand Island, NY) using QuantiTect SYBR Green PCR Kit (QIAGEN) for detection of IL-1β, TNFα and TLR4. Relative change in mRNA expression was calculated by use of the 2^−ΔΔCT^ values. GAPDH was used as an internal control. The primer sequences were as follows: for GAPDH, 5′-ACAGTCGCCGCATCTTCTT-3′ and 5′-CTTGATTTTGGAGGGATCTCGC-3′; For TNFα, 5′-GCCTCTTCTCATTCCTGCTTG-3′and 5′-CTGATGAGAGGGAGGCCATT-3′); for IL-1β, 5′GAGCACCTTCTTTTCCTTCATCTT-3, 5′TCACACACCAGCAGGTTATCATC-3′; for TLR4 5′-CAAGAACATAGATCTGAGCTTCAACCC-3′, 5′-CAAGAACATAGATCTGAGCTTCAACCC-3′. using thermocycler parameters of 95 °C for 5 minutes, followed by amplification of cDNA for 40 cycles of denaturation 95 °C for 15 seconds, annealing at 60 °C for 60 seconds and extension at 72 °C for 1 minute All reactions were done in triplicate using an Mx3005P QPCR System (Stratagene). The individual experiments were performed three times.

### Cytokine quantification by ELISA

4 corneas from each group (DMSO and SAHA) of the mice for the application of real-time PCR and ELISA, 8 corneas were collected from one experiment. For cytokine production, the mice corneas were dissected, placed in 0.5 ml reagent diluent, and homogenized using a Retsch MM 300 ball miller at 33 Hz for 4 min (Qiagen). Soluble protein extracts were then diluted, and TNFα and IL-1β production was measured by ELISA according to the manufacturer’s instruction (R&D Systems, Minneapolis, MN). The individual experiments were performed three times.

### Statistical analysis

Data is expressed as means ± SD and Student’s t-test was used for comparing the differences between two groups. A p-value < 0.05 was considered to be statistically significant.

The datasets generated during and/or analyzed during the current study are available from the corresponding author on reasonable request.
